# Use of Lentiviral Particles As a Cell Membrane-Based mFasL Delivery System for *In Vivo* Treatment of Inflammatory Arthritis

**DOI:** 10.3389/fimmu.2017.00460

**Published:** 2017-04-21

**Authors:** José M. Rodríguez-Frade, Anabel Guedán, Pilar Lucas, Laura Martínez-Muñoz, Ricardo Villares, Gabriel Criado, Dimitri Balomenos, Hugh T. Reyburn, Mario Mellado

**Affiliations:** ^1^Department of Immunology and Oncology, Centro Nacional de Biotecnología/CSIC, Madrid, Spain; ^2^Inflammatory and Autoimmune Diseases Group, Instituto de Investigación Hospital 12 de Octubre (i+12), Madrid, Spain

**Keywords:** FasL, lentiviral particles, apoptosis, caspases, rheumatoid arthritis

## Abstract

During budding, lentiviral particles (LVP) incorporate cell membrane proteins in the viral envelope. We explored the possibility of harnessing this process to generate LVP-expressing membrane proteins of therapeutic interest and studied the potential of these tools to treat different pathologies. Fas-mediated apoptosis is central to the maintenance of T cell homeostasis and prevention of autoimmune processes. We prepared LVP that express murine FasL on their surface. Our data indicate that mFasL-bearing LVP induce caspase 3 and 9 processing, cytochrome C release, and significantly more cell death than control LVP *in vitro*. This cytotoxicity is blocked by the caspase inhibitor Z-VAD. Analysis of the application of these reagents for the treatment of inflammatory arthritis *in vivo* suggests that FasL-expressing LVP could be useful for therapy in autoimmune diseases such as rheumatoid arthritis, where there is an excess of Fas-expressing activated T cells in the joint. LVP could be a vehicle not only for mFasL but also for other membrane-bound proteins that maintain their native conformation and might mediate biological activities.

## Introduction

Lentiviral vectors are very widely used in biological research, functional genomics, and gene therapy since they can mediate gene transfer into dividing and non-dividing cells both *ex vivo* and *in vivo*. Several examples of their use to correct genetic defects in have been described in both human disease (1–3) and animal models (4–7). Lentiviral particles (LVP) are generated through transient transfection of three plasmids in the human embryonal kidney (HEK293T) cells, a packaging, a transfer, and an envelope-encoding plasmid (8). As the LVP bud from the cell, membrane fragments bearing cellular proteins are incorporated into the LVP. For example, we found that CXCR4 is incorporated into virions that can be easily purified and maintain the ability to bind CXCL12 with native affinity (9). It has also been observed that several other chemokine receptors, when expressed in retroviral particles, can still bind antibodies and HIV-gp120 Env specifically (10), demonstrating the correct folding of these receptors in the LVP. Here, we addressed the hypothesis that the controlled incorporation of specific membrane glycoproteins into LVP could be utilized to generate nanoparticles able to modulate immune system function.

The Fas (CD95)/FasL (CD178) receptor/ligand interaction is a key factor in the induction of programmed cell death (11). Fas/FasL belong to the tumor necrosis factor (TNF)/TNF receptor (TNFR) family (12) and, like other members of the TNF family, biologically active FasL is a trimer (13). A FasL trimer recruits three Fas molecules, resulting in oligomerization of an intracellular death-inducing signaling complex (14) that through a cascade of several caspases triggers programmed cell death (11). This pathway is active during CTL-mediated killing of virus-infected or cancer cells (15) and in suppression/regulation of activated effector T cells (16, 17), among other processes. Fas-mutant (lpr) and FasL-deficient (gld) mice suffer autoimmune pathology due to dysregulated lymphoproliferative processes, indicating the relevance of this mechanism in the regulation of immune system homeostasis (18, 19).

Fas expression is observed in a variety of immune and non-immune cells (19), whereas FasL at the cell membrane (mFasL) is expressed by CD8^+^ cytotoxic T cells as well as a proportion of activated CD4^+^ T cells and NK cells. mFasL is processed by metalloproteases to generate soluble FasL (sFasL) that although still able to bind Fas, is much less efficient in killing target cells (20), consistent with the idea that clustering of preassembled Fas trimers is required for optimal apoptosis induction (21).

Here, we generated non-infectious lentiviral expressing murine FasL (FasL-LVP) and tested their efficiency in specific killing of Fas-expressing cells. Exposure of Fas-expressing cells to the FasL-LVP triggered activation of caspase 3 and subsequent apoptosis, as shown by annexin V (AV)-propidium iodide (PI) incorporation and cell cycle analysis, with no crosslinking requirement. FasL-LVP injection in the footpad of mice with established collagen-induced arthritis caused a reduction in paw inflammation, of cells infiltrating the tissue, as well as clear improvement in the clinical score compared to controls. Our *in vitro* and *in vivo* results indicate that administration of non-infective, non-replicative LVP expressing FasL could be a novel method for local treatment of inflammatory diseases. This pharmaceutical formulation avoids the requirements for FasL purification that might affect its conformation and/or activity and reduces side effects due to crosslinking requirements with anti-FasL antibodies.

## Materials and Methods

### Cells

HEK293T cells (human embryonic kidney cells) were obtained from American Type Culture Collection (ATCC CRL-11268, UK). The IL-3-dependent murine pro-B cell line, BaF/3, was obtained from German Collection of Microorganisms and Cell Cultures (DSMZ ACC300, Germany), and the murine pre-B lymphoma cell line L1.2 (22) was cultured in RPMI 1640 medium (BioWhittaker) supplemented with 10% FCS, 2 mM l-glutamine; for BaF/3, 10% conditioned medium of an IL-3-producing WEHI-3B cell line (DSMZ ACC26) was added to medium.

Thymus tissue from C57BL/6 mice (Harlan Laboratories, Inc., USA) was disaggregated with a potter homogenizer and the isolated thymocytes cultured in RPMI, 50 µM β-mercaptoethanol and 10 mM HEPES (pH 7.4). All cells were maintained at 37°C with 5% CO_2_.

### Generation of GPI-Linked FasL (FasL-GPI) Constructs

To prepare the construct encoding murine FasL attached to the membrane *via* a glycosylphosphatidylinositol (GPI) anchor the following oligonucleotides: mFasLFor—AGAGTCGACGCCACCATGCAGCAGCCCATGAATTAC; mFasLRev—GCAAGCTTAGAGCTTATACAAGCCGAAAAAGG; FasLFor—AGAGTCGACGCCACCATGCTGGGCATCTGGACCCTCCTACCTCTGGTTCTTACGTCTGTTGCTACACCCTC; mFasLGPIFor—ACGTCTGTTGCTACACCCTCTGAAAAAAAAGAGC; and mFasLGPIRev—GCAAGCTTGCCACCAGAGCTTGAACTGAGCTTATACAAGCCGAAAAAGG. DAFGPIFor—GCAAGCTTCCAAATAAAGGAAGTGGAACC; DAFGPIRev—GCATGCGGCCGCTAAGTCAGCAAGCCCATGGTTAC were used to amplify fragments of mFasL, murine DAF, and a linker sequence from mouse spleen cDNA, and then different combinations of these oligonucleotides were used to amplify overlapping fragments from the first round of PCR to generate a full length mFasL-GPI fragment that was cloned into the pBJ-Neo vector (23).

### Generation of LVP

Lentiviral expressing murine FasL (FasL-LVP) were produced by transient co-transfection of HEK293T cells with mFasL-GPI-LVTHM/GFP plasmid and, 24 h later, with PAX2 plasmid (Tronolab, Switzerland) at a 1:1 ratio using JetPEI (Polyplus-transfection; Illkirch, France). At 72 h posttransfection, cell supernatant was collected and centrifuged (30 min, 350 × *g*, RT) followed by ultracentrifugation in a Beckman SW55 rotor (2 h, 46,000 × *g*, 4°C) through a 20% sucrose cushion. The LVP-containing pellet was concentrated, resuspended in PBS, and stored in aliquots at −80°C. Control LVP were produced as above using HEK293T cells transfected with empty LVTHM/GFP and PAX2 plasmids (control HEK293T cells).

### Flow Cytometry Analysis

For cell staining, cells were incubated (3 × 10^5^ cells/well) with appropriate antibodies diluted in 50 µl staining buffer (PBS, 5% FCS, 1% BSA, 5 mM EDTA). After incubation (30 min, 4°C), cells were washed twice with staining buffer and analyzed in a Gallios cytometer (Beckman Coulter, CA, USA). Experiments were analyzed using FlowJo software (FlowJo, OR, USA).

Antibodies used include anti-mCD95-PE (BD Biosciences, CA, USA), -mCD95L-PE (BD Biosciences), and -CXCR4-biotin (R&D Systems, MN, USA). Streptavidin PE was from Beckman Coulter.

### Attachment of LVP to Latex Beads

To evaluate FasL-expressing LVP, we used flow cytometry of LVP coupled to latex beads. Aldehyde/sulfate latex beads (4 µm, 4% w/v; A37304, Invitrogen, ThermoFisher Scientific, MA, USA) were sonicated (5 min, RT) and mixed in a 1:3 v/v ratio with LVP (15 min, RT). PBS (1 ml) was added, and the final suspension was incubated (60 min, 4°C) with continuous rocking. Free reactive groups were blocked using glycine (100 µl, 1 M, 30 min, RT). Beads coupled to LVP were washed with PBS/BSA 0.5%. For FACS analysis, the pellet was resuspended in PBS staining buffer and stained with specific antibodies as described above.

### Cell Cycle Analysis

Thymocytes or BaF/3 cells (5 × 10^5^) were incubated (37°C, 5% CO_2_) with 10 µg/ml control LVP or FasL-LVP (6 h, 37°C). After washing with PBS, cells were resuspended in 50 µl detergent (DNA-Prep Reagent Kit, Beckman Coulter) containing 10 ng/ml PI (DNA-Prep Reagent Kit; 30 min, 37°C). Cell cycle phases were analyzed by flow cytometry. sFasL (2 µg/ml; Peprotech, NJ, USA) crosslinked with anti-6-His antibody (10 µg/ml, Sigma) was used as control.

### AV-PI Incorporation

Thymocytes, BaF/3, or L1.2 cells (5 × 10^5^) were incubated (37°C, 5% CO_2_) with 10 µg/ml control LVP or FasL-LVP (at different time points, 37°C). Cells were stained with AV-FITC and with PI following the manufacturer protocols (Aposcreen Annexin V apoptosis kit-FITC; Southern Biotech, AL, USA). All samples were analyzed in a Gallios cytometer. The number of early apoptotic (AV+/PI−) and necrotic/late apoptotic (AV+/PI+) cells was expressed as a percentage of total cells.

### Western Blot

Cells were lysed in 100 µl lysis buffer containing triethanolamine (20 mM, pH 8) and 2% digitonin, supplemented with protease inhibitors, aprotinin and leupeptin (20 µg/ml each), PMSF (1 mM), and sodium orthovanadate (10 µM). Lysis was carried out with agitation (30 min, 4°C). After centrifugation (15 min, 24,000 × *g*, 4°C), protein concentration in lysates was determined using BCA (Pierce, IL, USA). Thymocyte lysates (40 µg) and LVP (10, 20, and 40 µl) were resolved by 12% SDS-PAGE, transferred to nitrocellulose membrane (Bio-Rad, CA), blocked with 5% dry milk in TBS (60 min, RT, rocking), and incubated (ON, 4°C) with primary antibodies: anti-mFasL (BD Biosciences), -caspase 8 (Alexis, Switzerland), -caspase 3 (Cell Signaling, MA, USA), or -GFP (Invitrogen, CA, USA). After incubation with appropriate peroxidase (PO)-conjugated secondary antibody (Dako, Denmark), proteins of interest were detected by chemiluminescence (ECL, Pierce, IL, USA). ImageJ software was used for quantitation.

### Immunofluorescence

Cells were incubated with Mitotracker (250 nM, 30 min, 37°C, MitoTracker Green FM, ThermoFisher) and seeded (1.5 × 10^5^ cells, 30 min, 37°C) on microscope slides coated with poly-l-lysine (20 µg/ml, ON, 4°C, Sigma-Aldrich). Cells were fixed (PFA 4%, 10 min RT, Sigma-Aldrich), permeabilized (0.2% Triton X-100 in PBS, 10 min, RT), and blocked with blocking buffer (PBS, 1% BSA, 0.1% goat serum, 0.05% T20, 30 min, RT). Anti-cytochrome C antibody was added (1:200 dilution in blocking buffer, 45 min, RT, AbCam) followed by anti-mouse Alexa 647 (1:500 dilution in blocking buffer, 45 min, RT, Molecular Probes). After extensive washing with PBS, samples were incubated with DAPI (1:100, 20 min, RT, Sigma-Aldrich) and analyzed in an Olympus FV1000 confocal microscope with a 63× NA 1.4 oil objective.

### Induction and Assessment of CIA

Two-month-old DBA/1J mice were immunized intradermally at the tail base with an emulsion of chicken type II collagen (CII) in Freund’s complete adjuvant (24). Arthritis was assessed daily by scoring each limb on a 0–4 scale, where 0 = normal, 1 = erythema and mild swelling confined to the tarsals or ankle joint, 2 = erythema and mild swelling extending from the ankle to the tarsals, 3 = erythema and moderate swelling extending from the ankle to metatarsal joints, and 4 = erythema and severe swelling encompass the ankle, foot and digits, or ankylosis of the limb, yielding a maximum score of 16 per mouse. Limbs with a score >2 (Cs_0_) were inoculated on day 0, 2, 4, 6, and 9 in the footpad with FasL-LVP or LVP (30 µg/ml, 50 µl) and the clinical score re-evaluated (Cs_t_). As control, unaffected limbs were treated in parallel with FasL-LVP or LVP (30 µg/ml, 50 µl).

At the end of the experiment, the score was monitored, and the paws were removed, fixed in 4% formalin, decalcified with 10% EDTA, and paraffin-embedded. Sections (4 µm thick) were stained with hematoxylin and eosin or alkaline phosphatase.

### Enzyme-Linked Immunoassay

Microtiter plates were coated with chicken type II collagen (5 µg/ml; 90 min, 37°C). After blockade of remaining protein-binding sites with 0.5% BSA in PBS, plates were incubated with serial dilutions of serum samples of immunized DBA/1J mice, followed by PO-labeled subclass-specific rabbit anti-mouse antisera. When needed, LVP- and FasL-LVP-coated microtiter plates (1 µg/ml) were incubated with serial dilutions of serum samples of mice injected with LVP in the footpad (time 0 and 14 days after treatment), followed by PO-rabbit anti-mouse IgG (Dako) antisera.

### Statistical Analysis

Results were analyzed with Prism 5.0 (GraphPad Software; ***p < 0.0001, **p < 0.001, *p < 0.05). We used one-way ANOVA analysis and Tukey’s multiple comparison test. Data are shown as mean ± SEM.

## Results

### LVP Express FasL at the Particle Surface

**Figure 1:**
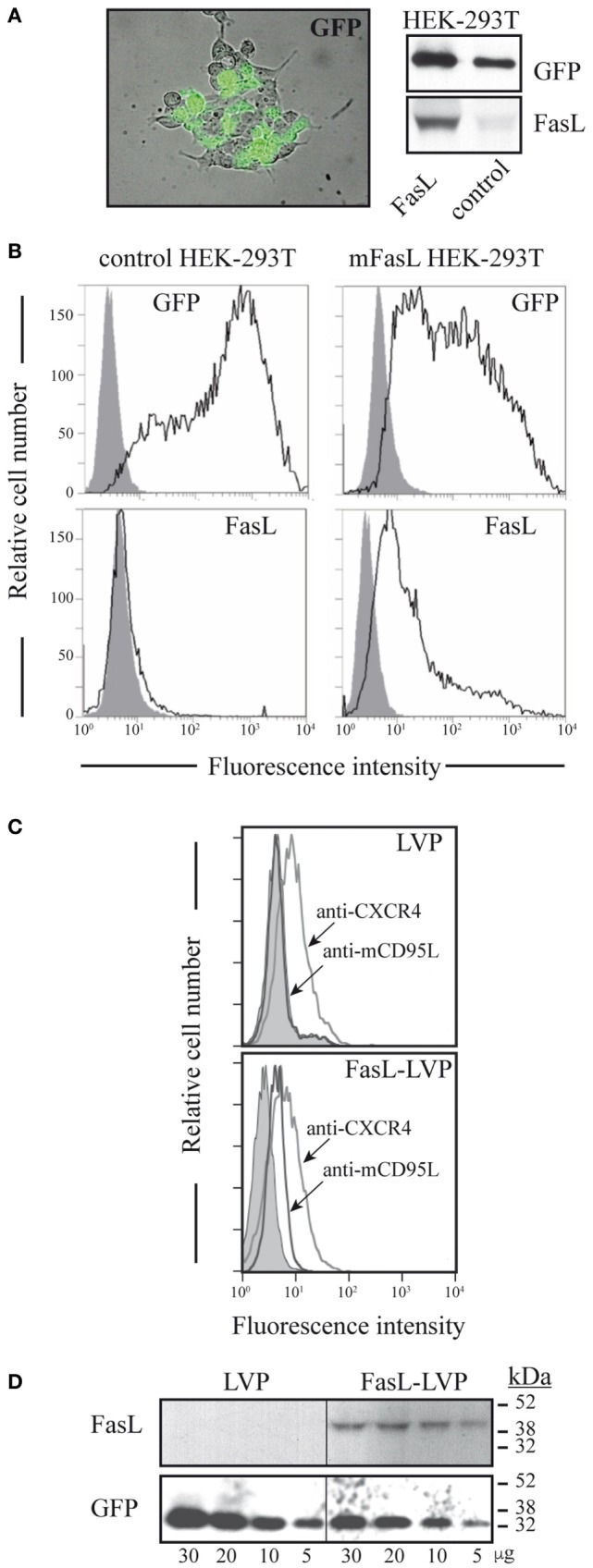
**FasL expression at the surface of lentiviral particles (LVP). (A)** HEK293T cells transfected with GPI-linked FasL or control plasmid were analyzed by fluorescence microscopy and Western blot and **(B)** by flow cytometry using specific anti-mFasL antibodies. GFP expression was analyzed as a control of cell transfection. One experiment is shown of five performed. The gray bell-shaped curve indicates detection in untransfected HEK293T cells. **(C)** Flow cytometry analysis of control and mFasL expression in LVP coupled to latex beads. A representative experiment is shown of five performed. **(D)** Western blot analysis of mFasL- and control LVP using specific anti-GFP and -mFasL antibodies. A representative experiment is shown of more than five performed.

We transiently transfected HEK293T cells with PAX2, pGEM-GFP and FasL-GPI-LVTHM/GFP-murine, or empty LVTHM/GFP plasmids (control). Transfection efficiency was controlled by assessing GFP expression *via* fluorescence microscopy, Western blot and flow cytometry, whereas flow cytometry and Western blot analysis using anti-FasL mAb confirmed specific expression of FasL in cells transfected with FasL-GPI-LVTHM/GFP (Figures 1A,B). Flow cytometry analysis of LVP, isolated by centrifugation through a sucrose pellet, using specific mAb showed that FasL could be detected on the surface of LVP obtained from cells transfected with FasL-GPI (FasL-LVP) (Figure 1C, lower panel), but not on LVP obtained from HEK293T cells transfected with a control vector (LVP) (Figure 1C, upper panel). Both types of particles (FasL-LVP and LVP) expressed similar levels of CXCR4, a receptor endogenously expressed by HEK293T cells (Figure 1C). To confirm FasL expression, detergent extracts of FasL-LVP and LVP were evaluated by Western blot using specific anti-FasL mAb (Figure 1D, upper panel). GFP expression in FasL-LVP and control LVP was analyzed as a loading control and was also used to normalize the number of control and FasL-LVP used in subsequent assays (Figure 1D, lower panel).

### mFasL-LVP Are Fully Functional *In Vitro*

**Figure 2:**
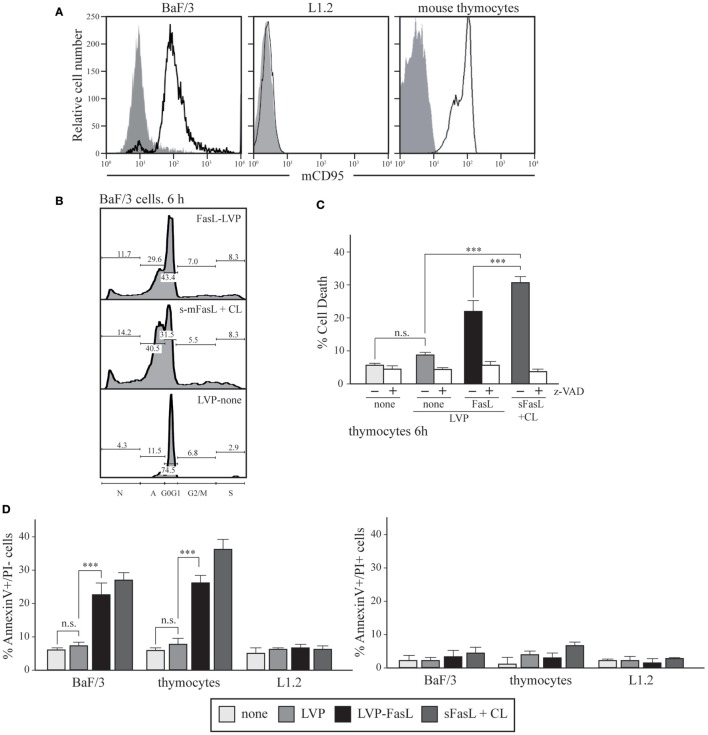
**FasL-LVP mediated induction of cell death**. **(A)** Flow cytometry analysis of Fas (CD95) expression in BaF/3; L1.2 cells and murine thymocytes. An experiment is shown of three performed. **(B)** Cell cycle analysis using propidium iodide (PI) incorporation and flow cytometry analysis of BaF/3 cells treated with crosslinked soluble FasL (sFasL), mFasL, and control lentiviral particles (LVP) for 6 h. Gates defining the different cell cycle phases are indicated. The figure shows one experiment of more than five performed. The percentages of cells in each cell cycle phase as well as those for cell apoptosis are shown. **(C)** Cell cycle analysis using PI incorporation and flow cytometry analysis of murine thymocytes, untreated or pretreated with Z-VAD and untreated or treated with crosslinked sFasL, mFasL, or control LVP for 6 h. The figure shows the percentage of apoptotic cells; data are shown as mean ± SD of six independent experiments (***p < 0.0001). **(D)** Apoptosis of murine thymocytes, BaF/3, and L1.2 cells treated with FasL- or control LVP (4 h). The number of early apoptotic (AV+/PI−) and necrotic/late apoptotic (AV+/PI+) cells was expressed as a percentage of total cells. Results of three independent experiments are shown as mean ± SD (***p < 0.0001).

Ba/F3 cells constitutively express Fas at the cell surface, which can be upregulated by IL-3 treatment (25) (Figure 2A, left). To test the ability of FasL-LVP to trigger cell apoptosis, Ba/F3 cells (10^6^ cells/ml) were plated in RPMI medium supplemented with 5% FCS and IL-3, and exposed to serial dilutions of FasL-LVP, control LVP, or sFasL plus crosslinker as a positive control. Cell cycle analysis by PI incorporation and flow cytometry showed that treatment with FasL-LVP significantly increased cell death compared to control (Figure 2B).

To test whether FasL-LVP treatment could also induce the death of primary cells, we used thymocytes, as they also express Fas at the cell surface (Figure 2A, right). Thymocytes isolated from 3-month-old C57BL/6 mice were treated with different amounts of FasL-LVP, LVP, or sFasL plus crosslinker for various times (2, 4, 6, 8, or 24 h) and analyzed as above. FasL-LVP significantly increased cell death compared to control LVP, with a maximum effect after 6 h treatment (Figure 2C). Pretreatment of cells with the pan-caspase inhibitor Z-VAD (R&D) blocked FasL-LVP-mediated apoptosis (Figure 2C), suggesting caspase involvement in the cell death process activated by exposure to FasL-LVP.

These results were confirmed by determining AV-PI incorporation in thymocytes, and Ba/F3 cells treated with FasL-LVP or control LVP. As an additional control of specificity, FasL-LVP did not trigger apoptosis of L1.2 cells (Figure 2D). Murine pre-B L1.2 cells do not express Fas at the cell surface, as shown by flow cytometry with anti-mCD95-PE mAb (Figure 2A).

**Figure 3:**
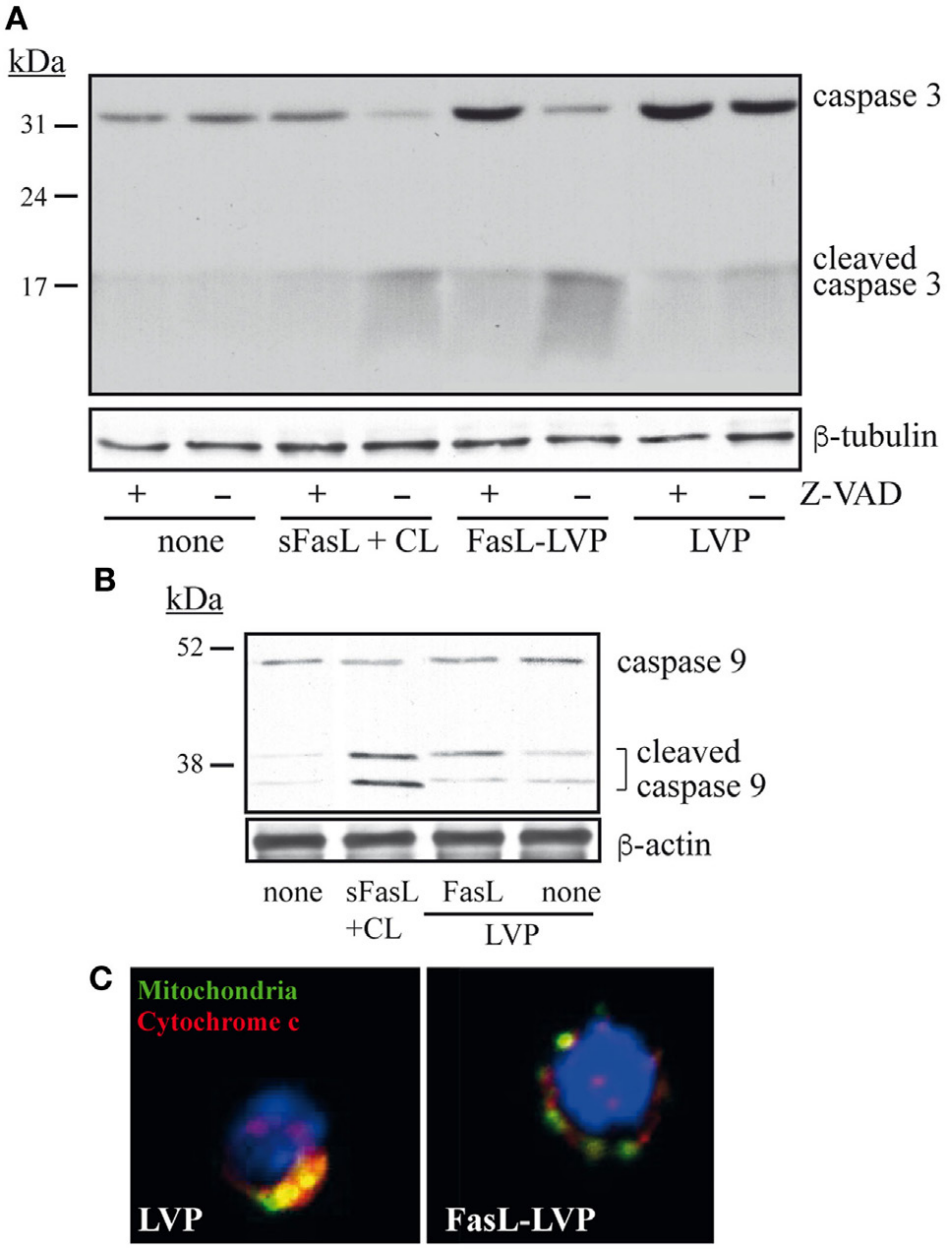
**Induction of caspase processing and cytochrome C release by FasL-LVP**. **(A)** Western blot analysis of caspase 3 processing in murine thymocytes, alone or pretreated with Z-VAD and untreated or treated with crosslinked soluble FasL (sasL), FasL- and control lentiviral particles (LVP) for 6 h, using specific antibodies. An experiment is shown of three performed. **(B)**. Western blot of caspase 9 processing in murine thymocytes as in panel **(A)**. An experiment is shown of three performed. **(C)** Immunofluorescence analysis of cytochrome c release using specific antibodies and murine thymocytes treated with FasL- or control LVP for 6 h. One experiment is shown of five performed.

FasL binding to Fas receptor leads to the formation of the DISC (death-inducing signaling complex), autoprocessing of the initiator procaspase 8 and activation of the effector procaspase-3, which triggers the extrinsic pathway of apoptosis (26) and/or cytochrome C release from mitochondria and caspase 9 activation (intrinsic pathway). Processing of procaspase 8 was not detected in any of these experiments (not shown); nevertheless, exposure to FasL-LVP specifically induced cleavage of caspase 3 in thymocytes incubated for 6 h with FasL-LVP (Figure 3A). FasL-LVP-induced cleavage of caspase 3 was blocked when thymocytes were pretreated with the pan-caspase inhibitor Z-VAD (Figure 3A), indicating the specificity of the effect. Cleavage of caspase 9 was also observed in these experiments (Figure 3B). Moreover, rapid, specific release of cytochrome C, indicative of mitochondrial damage, was detected when thymocytes were treated with FasL-LVP, but not LVP controls (Figure 3C). Overall, these data showing involvement of both caspases 3 and 9 and mitochondrial damage in the cell death of thymocytes treated with FasL-LVP suggests that exposure to these particles triggers both the extrinsic and intrinsic pathways of apoptosis.

### Local Administration of FasL-LVP Reduces Paw Swelling in a Murine Model of Arthritis

**Figure 4:**
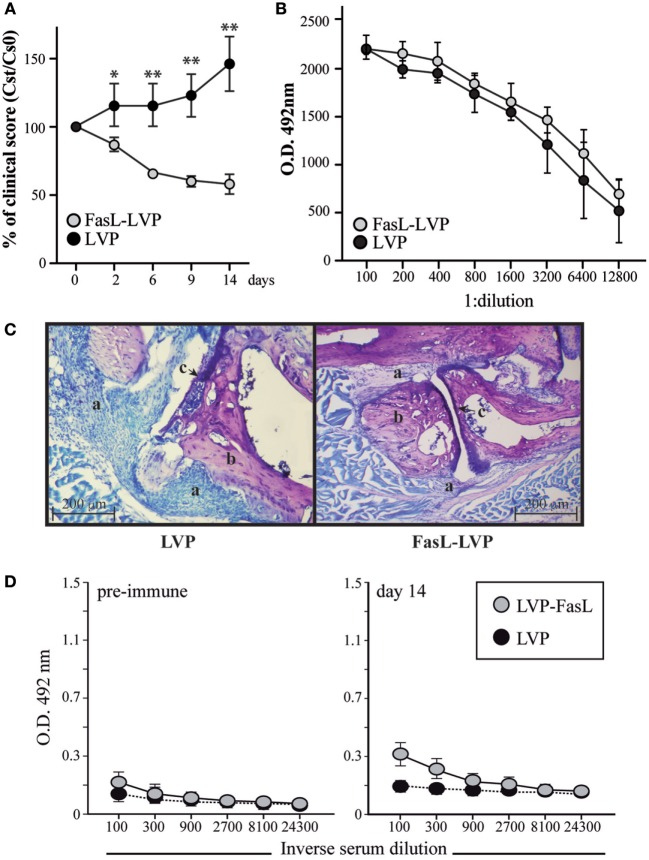
**Effect of local administration of FasL-LVP in a murine model of arthritis**. **(A)** Evolution of the paw swelling score after treatment with FasL- or control LVP (Cs_t_), expressed as a percentage of the initial score (Cs_0_) (*n* = 12; **p < 0.001, *p < 0.05). **(B)** Levels of total anti-collagen II IgG (micrograms per milliliter) were measured in mouse sera by ELISA at the end of the experiment (day 14 posttreatment). **(C)**. Eosin–thiazin staining of representative sections of ankle joints of the mice in panel **(A)** at day 14 posttreatment with lentiviral particles (LVP) (left) or FasL-LVP (right). The figure shows decreased cell infiltration (a), bone erosion (b), and cartilage destruction (c) in FasL-LVP-treated mice. Original magnification 10×. **(D)** Levels of total anti-LVP IgG were measured in mouse sera by ELISA on days 0 and 14 after LVP and FasL-LVP treatments.

The collagen-induced arthritis model in DBA/1J mice was used to assess the potential therapeutic utility of FasL-LVP administration, since Fas expression is enhanced on activated lymphocytes and the immunopathogenesis in this model involves both T-cell and B-cell-specific responses to collagen (27). Arthritis was induced by subcutaneous injection of collagen type 2 into 2-month-old DBA/1J mice. On appearance of the first signs of CIA (score ≥2, day 0), arthritic mice received four injections on days 0, 2, 4, and 6 of either FasL-LVP or LVP (30 µg/ml, 50 µl) in the inflamed paw (left or right limbs, respectively). While disease progressed in LVP-treated mice, a clear reduction in paw swelling (*p* < 0.01) (Figure 4A) was observed in FasL-LVP-treated animals. This effect was observed as early as 2 days after initiation of treatment and reached maximum at 14 days after the initial inoculation. Treatment with neither FasL-LVP nor LVP promoted any changes in non-inflamed paws. The reduced severity of disease in FasL-LVP-treated mice was not associated with changes in anti-collagen type II antibody levels (Figure 4B), suggesting that the effect is confined mainly to the administration site. Histological analysis of paws from both FasL-LVP- and LVP-treated mice showed that the inflammation and pannus parameters differed clearly between these groups. Similarly, the damage observed in cartilage and bone in control mice was absent in those treated with FasL-LVP (Figure 4C). We nonetheless detected a small but sustained immune response to LVP in treated mice (Figure 4D). The use of the human HEK293T cell line to prepare the LVP might underlie this side effect.

## Discussion

As they bud from the membrane of the cell, LVP incorporate components of the plasma membrane, including embedded proteins in their lipid environment, into the virus particle (28). Here, we show that this property can be exploited to develop virus-like particles that can subsequently modulate cell activation both *in vitro* and *in vivo*.

The Fas molecule belongs to a subset of the TNFR family called death receptors, which can trigger apoptosis by inducing caspase activation. Ligation of Fas triggers apoptosis in activated T cells; this mechanism is involved, for example, in regulation of the clonally expanded T cell population after antigen clearance (11). Fas-mediated apoptosis can also be activated in other, non-immune, cell types including the death of neurons after withdrawal of growth factors, of fibroblasts expressing c-Myc, of thyroid cells and even of endothelial cells (29–32), which suggests that Fas can be considered a target for therapeutic intervention in several diseases.

We engineered and transiently expressed a chimeric FasL-GPI in HEK293T cells and used them to produce LVP that express FasL, but lack viral envelope proteins. Replacement of C-terminal transmembrane domains by GPI-addition signal peptides allows expression of the proteins on the plasma membrane and provides stable association of the construct with the lipid bilayer (33, 34). Plasmids encoding viral envelope proteins, i.e., VSV-G (glycoprotein of the vesicular stomatitis virus), were not used in the viral particle preparation since VSV-G expression facilitates virus dissemination, promoting a range of non-specific changes that could obscure the specific effects of treatment with FasL (35).

*In vitro*, the virus-like particles induced cell death in Fas-positive cells, including the B cell line, BAF/3 and primary thymocytes. The absence of apoptosis in L1.2 cells, which do not express cell surface Fas, and in cells treated with control LVP showed that this effect is specific; it indicated that FasL-LVP-induced cell death is mediated by the FasL expressed on the particles and not by other components expressed at the membrane or by other LVP components. In this context, when VSV-G pseudotyped LVP were used in similar experiments, increased cell death was observed independently of FasL, probably due to the ability of these LVP to infect cells (36).

FADD-mediated activation of the proteolytic activity of caspase 8 is essential for Fas-induced apoptosis in many cell types (37–39); nonetheless, caspase 8 cleavage was not observed in our experiments. We demonstrated processing of caspase 3 and caspase 9 as well as cytochrome C release from mitochondria, which suggests FasL-mediated activation of both the extrinsic and intrinsic apoptosis pathways. It is possible that in our experimental conditions, modest activation of caspase 8 was undetected; indeed, a Fas signaling pathway involving slowly activated caspase 8 and mitochondrial damage has been reported (40). This pathway involves lower levels of activated caspase 8 which are still sufficient to process the pro-apoptotic protein Bid. Translocation of Bid to the mitochondrial membrane induces oligomerization of Bcl-2, Bak, and Bax, which promote cytochrome release and finally caspase 9 activation (41). Nevertheless, the possibility of FasL-LVP-mediated activation of a caspase 8-independent apoptosis mechanism cannot be excluded. Caspase-independent activation of JNK leading to cell death has been reported (42), as has Fas-mediated cell death that is not blocked by specific caspase 8 inhibitors (43). New experiments are required to clarify this issue. Whatever the mechanism, the data presented here clearly show that LVP displaying FasL can induce apoptosis in Fas-expressing cell lines and primary cells *in vitro*.

Although apoptotic cells are rarely observed in RA tissues *in vivo* (44), probably due to intra- and extracellular antiapoptotic processes (45, 46), high Fas/FasL levels are present in the synovium of RA patients (44). Since apoptosis induced by anti-Fas antibody or by gene transfer of FasL ameliorates arthritis in the experimental murine model of collagen-induced arthritis, probably *via* the induction of apoptosis of T cells, macrophages, and synoviocytes (47–49), we tested the use of our LVP as therapy in this model. Local injection of FasL-LVP into the inflamed footpad specifically reduced paw inflammation. This effect was restricted to the injected paw, as no improvement of inflammation was noted in the contralateral hind paw that received the injection of control LVP. In addition, no effect was observed on anti-collagen type II antibody levels. These data indicate that the injected LVP did not disseminate outside the injection site. Histological analysis reflected a significant reduction of cell infiltration in joints treated with FasL-LVP compared to controls. The simplest interpretation of these experiments is that the FasL-LVP induces death of the activated (Fas-expressing) lymphocytes and macrophages that mediate the autoimmune process. We also detected low anti-LVP antibody levels in the sera of treated mice, which indicated that LVP treatment might activate a slight immune response to the viral particles, which could decrease treatment efficiency. The use of human HEK293T cells to generate the LVP for use in a murine system might underlie this effect. LVP generation in compatible cells should reduce this side effect. LVP have been widely used for human gene therapy for many years, and several strategies have been developed to avoid, suppress, or manipulate the immune response and promote immune tolerance to the LVP (50).

The use of FasL-LVP as a therapeutic strategy has a number of advantages compared to the use of sFasL. It is suggested that non-apoptotic, Fas-mediated signals can promote chronic inflammatory arthritis. For instance, DBA1/lpr mice, which are defective in Fas signaling, are less susceptible to CIA (51). Indeed, sFasL is increased in synoviocytes of RA patients compared to those suffering from OA, and sFasL levels are higher in patients with severe RA compared to those with mild disease, indicating that sFasL may be an exacerbating factor in RA (52). sFasL, cleaved from cell membranes by metalloproteases, contributes to Fas downregulation, thus reducing Fas-mediated apoptosis in RA synovial cells and perpetuating the disease (53). The construct used in the preparation of FasL-LVP lacks the proteolysis site found in membrane FasL, which minimizes the possibility of sFasL production. Another advantage of this construct is that the intrinsic structure of the FasL displayed on the virus-like particle is likely very similar to that on the cell membrane, rendering crosslinking with antibodies unnecessary. The use of monoclonal antibodies to crosslink sFasL is associated with liver toxicity (54), whereas a synthetic human hexameric FasL (APO010, Topotarget) promotes human glioma cell death and prolongs survival in tumor-bearing mice with no need for crosslinkers (55). *In vivo*, Fas expression is enhanced after lymphocyte activation; we also demonstrated that FasL-LVP, presumably by inducing the death of activated cells, can be used to treat autoimmunity. Our *in vitro* and *in vivo* results indicate that the administration of non-infective, non-replicative LVP expressing FasL could be a novel method for local treatment of inflammatory diseases.

## Ethics Statement

All animal experiments were performed in accordance with national and European regulations and were approved by the Animal Experimentation Committees at the CSIC and the Dirección General de Medio Ambiente de la Comunidad de Madrid (PROEX 188/14).

## Author Contributions

JMR-F and MM designed research; JMR-F, AG, PL, LM-M, RV, GC, and HR performed research; GC, DB, and HR contributed new reagents/analytic tools; JMR-F, RV, and MM analyzed data; and JMR-F, HR, and MM wrote the paper.

## Conflict of Interest Statement

The authors declare that the research was conducted in the absence of any commercial or financial relationships that could be construed as a potential conflict of interest.
